# Fluctuating Asymmetry in *Menidia beryllina* before and after the 2010 Deepwater Horizon Oil Spill

**DOI:** 10.1371/journal.pone.0118742

**Published:** 2015-02-25

**Authors:** Savannah Michaelsen, Jacob Schaefer, Mark S. Peterson

**Affiliations:** 1 University of Southern Mississippi, Department of Biological Sciences, Hattiesburg, MS, 39406, United States of America; 2 Southeastern Louisiana University, Department of Biological Sciences, Hammond, LA, 70402, United States of America; 3 University of Southern Mississippi, Department of Coastal Sciences, Ocean Springs, MS, 39564, United States of America; Dauphin Island Sea Lab, UNITED STATES

## Abstract

Assessing the impacts of the Deepwater Horizon oil spill with a dependable baseline comparison can provide reliable insight into environmental stressors on organisms that were potentially affected by the spill. Fluctuating asymmetry (small, non-random deviations from perfect bilateral symmetry) is an informative metric sensitive to contaminants that can be used to assess environmental stress levels. For this study, the well-studied and common Gulf of Mexico estuarine fish, *Menidia beryllina*, was used with pre and post-oil spill collections. Comparisons of fluctuating asymmetry in three traits (eye diameter, pectoral fin length, and pelvic fin length) were made pre and post-oil spill across two sites (Old Fort Bayou and the Pascagoula River), as well as between years of collection (2011, 2012)-one and two years, respectfully, after the spill in 2010. We hypothesized that fluctuating asymmetry would be higher in post-Deepwater Horizon samples, and that this will be replicated in both study areas along the Mississippi Gulf coast. We also predicted that fluctuating asymmetry would decrease through time after the oil spill as the oil decomposed and/or was removed. Analyses performed on 1135 fish (220 pre and 915 post Deepwater Horizon) showed significantly higher post spill fluctuating asymmetry in the eye but no difference for the pectoral or pelvic fins. There was also higher fluctuating asymmetry in one of the two sites both pre and post-spill, indicating observed asymmetry may be the product of multiple stressors. Fluctuating asymmetry decreased in 2012 compared to 2011. Fluctuating asymmetry is a sensitive measure of sub lethal stress, and the observed variability in this study (pre vs. post-spill or between sites) could be due to a combination of oil, dispersants, or other unknown stressors.

## Introduction

Fluctuating asymmetry (FA, small non-random deviations from perfect bilateral symmetry) has been used in a wide variety of taxa as an indicator of exogenous stressors[1,2]. The development of left and right sides of bilateral traits can be viewed as replicates of the same structure [3,4]. In the absence of stress or other external inputs, development occurs along pre-determined paths potentially resulting in identical left and right sides-perfect symmetry [2]. However, organisms rarely develop with perfect symmetry as developmental noise (due to a variety of inherently stochastic processes) results in low levels of asymmetry [3,5]. Environmental stressors that perturb developmental pathways (mechanisms that serve to stabilize development) will further increase asymmetry [2]. Fluctuating asymmetry has been shown to be a sensitive indicator of contaminants, and yield reliable and inexpensive data on sub-lethal stress in wild populations [6], especially fishes [5,7]. One distinct advantage of using FA as an indicator of environmentally induced stress is that museum specimens can be utilized to establish baseline levels of FA before a particular disturbance of interest [8]. Pre-disturbance museum specimens can be directly compared with post-disturbance collections provided: 1) the sampling and treatment of individuals is the same (i.e. individuals are randomly sampled from the environment using the same gear in the same habitats across all collections) and 2) storage of specimens in the museum does not alter any of the traits of interest [8].

On April 20, 2010 the Deepwater Horizon (DWH) oil spill began and over the next 89 days leaked oil into the Gulf of Mexico (GOM) [9–11]. Reports of damage in the media and scientific literature ranged from direct mortality of charismatic species [12] to changes in ecosystem services [13–15], developmental anomalies [16], lesions [17] and physiological effects [18,19]. Other research has concluded that recruitment and patterns of fish assemblage structure in the 2010 year class were normal and that attention should focus on delayed responses from longer term exposure [20,21].

A challenge in assessing impacts is that reliable baseline data (pre DWH in this case) on pertinent response variables are often absent, leaving post DWH impact data without meaningful comparison [12,22]. Approaches to this problem include designating sites as non-contaminated “controls” because of reported low levels of oilage, or doing controlled experiments in a common garden. Designation of sites as non-contaminated is problematic for a spill the size and scope of DWH as this makes tenuous assumptions of: 1) reliable detection in complex ecosystems, 2) availability of pertinent contamination data, and 3) no dispersal of individuals among putative contaminated and non-contaminated sites. Overall, the lack of baseline data leaves reports of biologic effects due to DWH open to criticism as flawed (contaminated control sites), anecdotal (no control sites) or lacking external validity (common gardens). The use of museum specimens has the potential of allowing for direct pre and post DWH comparisons as long as the response variables can be accurately quantified in both groups.

Oil spill impacts are expected to be highest in coastal habitats where oil accumulates and mixing is facilitated by wave action [22]. The Inland Silverside, *Menidia beryllina*, occurs in estuaries, coastal rivers and bays throughout the GOM, Mississippi River basin and as far north as Massachusetts [23]. It is one of the most abundant and widely studied fish in northern GOM habitats that could have been affected by DWH. Due to its abundance in coastal habitats, *M*. *beryllina* has been used as a model for a variety of oil toxicology studies [24–26] that have demonstrated lethal and sublethal (morphological and physiological) effects of oil exposure. The abundance of *M*. *beryllina* also ensures that the life history is well documented [27,28] and that there is an abundance of specimens available for study in natural history collections. *Menidia beryllina* spawn in March through July, grow and mature quickly, and are thought to have a one or two year lifespan [28–30]. As a result, large adults sampled in the spring and summer of 2011 are expected to have been born and developed during the peak of DWH oil presence along the northern GOM coast. Specimens collected in 2012 were most likely not born during the 2010 cohort, and therefore expected to show lower FA due to less direct oil exposure. *Menidia beryllina* are thought to show high site fidelity with movement restricted to within estuaries [31].

The purpose of this study is to use FA as a metric of environmental stress due to oil contamination from DWH. We measured FA in *M*. *beryllina* collected before DWH to establish baseline levels with which we compared FA in post DWH fish collected at the same locations using the same sampling gear. We hypothesize that levels of FA will be higher in post DWH samples, and that this response will be the same in two replicate areas along the Mississippi coast. We also quantified FA in fish collected one (2011) and two (2012) years post DWH to test the hypothesis that FA would decrease through time after DWH as oil decomposed or was removed through cleanup efforts and polycyclic aromatic hydrocarbons in tissues decreased [17].

## Methods

### Fish Collection

**Fig 1:**
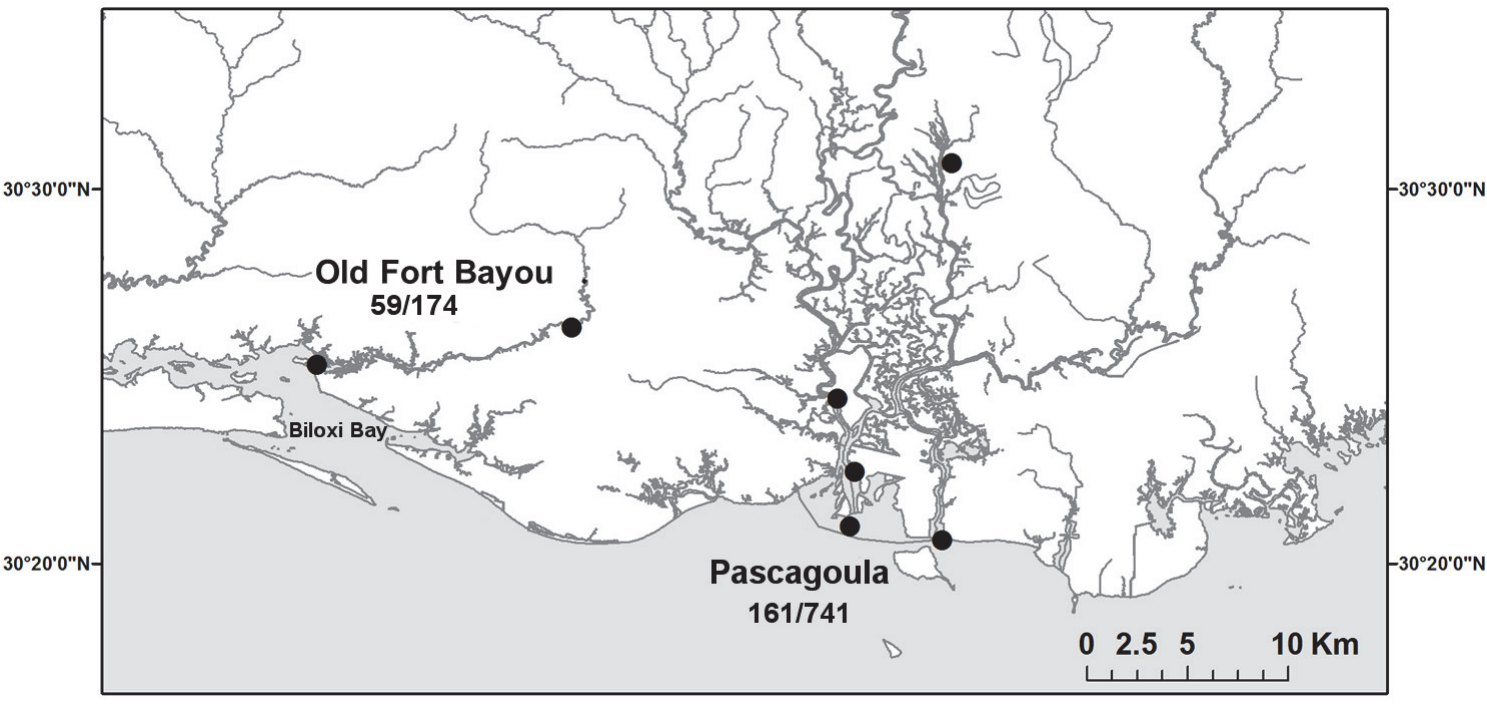
Map of the study are with the seven sites shown. Numbers indicate the total sample size (pre/post DWH) for Old Fort Bayou (OFB) and Pascagoula (PAS).

We collected fish from two sites in Old Fort Bayou (OFB) and five sites in the lower Pascagoula River (PAS) (Fig. 1). These sites were chosen because earlier collections had been made for previous studies in the same locations [32–34], and collected specimens of *M*. *beryllina* were deposited into the University of Southern Mississippi Ichthyological Collection. Pre DWH collections were made during earlier, separate projects in 1985–1986 (for both OFB sites) and 2002–2008 (for all five PAS sites). Post DWH collections for this study were made monthly at all seven sites from May 2011 through November 2012. Both pre and post DWH fish were collected as part of broad community wide sampling that targeted all available habitats with a seine. Thus, *M*. *beryllina* were not targeted in any of the sampling and thus represent a random selection of individuals from the population. All fish were collected by seine (0.32 cm mesh) and fixed in 10% formalin before being transferred to 70% ethanol, cataloged, and deposited into the museum. Post DWH fish remained in ethanol for a minimum of two months before measurements were made. From each collection, we randomly selected a maximum of 20 individuals that were a minimum of 30 mm in standard length (mean 49.1 mm, maximum 86.9 mm) for FA measurements. Thirty mm was chosen as the minimum length to minimize measurement error that would arise from variance in repeated measures of small traits (see below). *Menidia beryllina* are thought to reach sexual maturity as soon as four to five months after birth [28], at which time they are expected to be close to 30mm. Methods of collection and preservation of fish were approved by the University of Southern Mississippi IACUC (protocol 10100107). A sampling permit (06022011) was issued by the Mississippi Department of Marine Resources.

Biloxi Bay and the Pascagoula River estuary are about 180 km north of the DWH spill site. There was documented surface (http://gomex.erma.noaa.gov/: National Oceanic and Atmospheric Administration satellite imagery) or beached oil (SCAT: shoreline cleanup and assessment technique) throughout the Mississippi coast [35]. The NOAA “maximum observed oiling” data classified shoreline habitat in both Biloxi and Pascagoula Bay as “no oil observed” to “moderate” while the barrier islands directly offshore were classified as “moderate” or “heavy”. Both models [35] and satellite imagery indicated oil near sites in both bays in the spring and summer of 2010. While precise measures of oil contamination would be informative, they are not particularly useful without corresponding information on the movements of *M*. *beryllina* during this period. Since we were not able to assign specific contamination levels to individual collections of fish, we regarded all post DWH fish in these collections as exposed for comparison to pre DWH fish representing the baseline.

### Quantifying FA

We used digital calipers (Mitutoyo series 500; Aurora, Illinois) to measure left and right sides of three bilateral traits: eye diameter (eye), length of longest pectoral fin ray (pectoral) and length of longest pelvic fin ray (pelvic). These three traits are commonly used in FA studies, and they have been shown to be effective in assessing FA specifically in fish [5,36–38]. Each fish was measured twice on separate occasions in order to properly account for measurement error (ME) in analyses [5,7,39]. After the second measurement, we measured standard length (SL, mm) and weighed the fish (wet weight, g). We used published age and growth data for *M*. *beryllina* [27,28] to construct a von Bertalanffy growth model to estimate a time of birth for all post DWH collected fish [40] that were then classified as belonging to the 2010, 2011 or 2012 year classes. We used Fulton’s condition index (1000*mass/SL^3^, condition) as an assay of individual fitness [41,42].

Individual signed FA was quantified as the difference between left and right measures divided by mean trait size to control for size effects and potential directional asymmetry [8,43]. Unsigned FA was calculated as the absolute value of signed FA. FA values were tested for normality (signed), directional asymmetry (signed) and used for visualization of the data (unsigned). We used Grubb’s test to identify and eliminate outliers (any individual that was an outlier in any of the three signed FA measures)[44]. The final dataset included FA measurements on 1,135 fish: 220 pre and 915 post DWS (Fig. 1). While there were pre and post DWH samples from all 7 sites, sample sizes varied considerably (ranging from 22 to 240 at individual sites), reducing power for tests among sites. Thus, we pooled sites within OFB and PAS to test for location effects (sample sizes in Fig. 1) and included individual site as a random effect in models. We used mixed model repeated measures ANOVA (with side being fixed, and individual fish and site random repeated factors) to partition measurement error [7,39] and test for differences in FA among groups of fish (pre vs. post DWH, OFB vs. PAS and between post DWH years). The same models were used separately on each of the three FA traits. To assess the significance of individual terms while controlling for measurement error, we used maximum likelihood ratio tests of nested models (comparing models with and without the term of interest). All analyses were performed in R [45].

## Results

There were no post DWH individuals estimated to have been part of the 2009 year class. A small number of individuals (n = 35) were estimated to have been born mid-2010 (after DWH). However, there were not enough of these fish for reliable analysis so 2010 fish were grouped with the 2011 age class. The final post DWH dataset included 735 fish from 2010–2011 and 180 from 2012.

**Fig 2:**
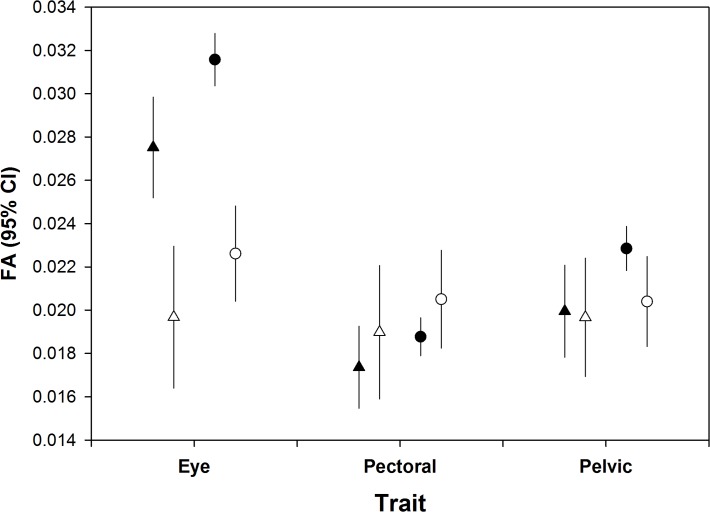
Mean unsigned FA (95% confidence intervals) for each trait in pre (open symbols) and post (closed symbols) DWH samples by location.

**Fig 3:**
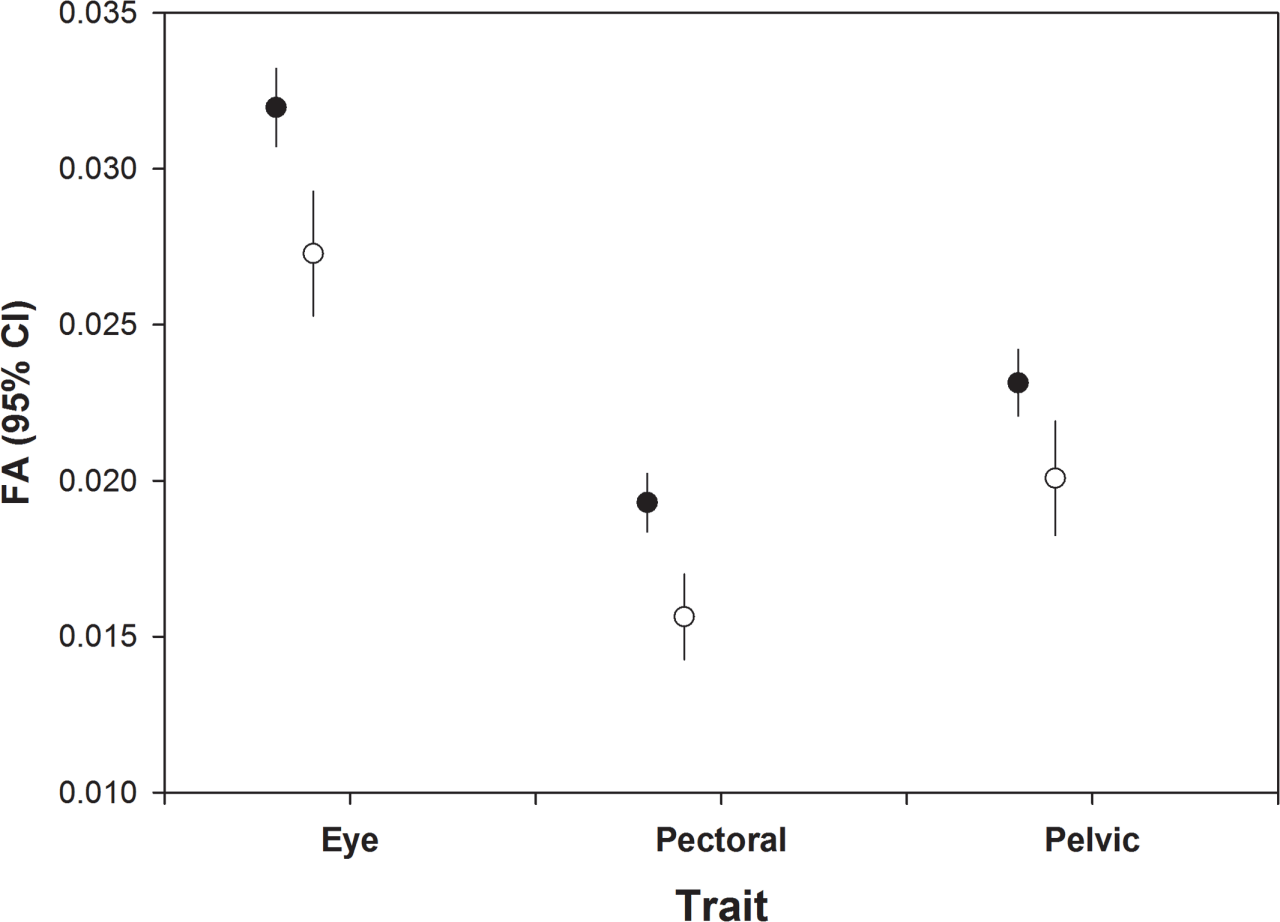
Mean unsigned FA (95% confidence intervals) for each trait by year in post DWH samples, pooling location.

Measurement error ranged from 6.2 to 8.8%, and there was no indication of directional asymmetry (Table 1). None of the three FA measures were significantly correlated with Fulton’s condition index (eye: F = 3.51, P = 0.06, pectoral: F = 0.61, P = 0.434, pelvic: F = 0.27, P = 0.61). FA was higher in post DWH than pre DWH fish in the eye, but not in the pectoral or pelvic fins (Table 2, Fig. 1). In all three traits, the trend was for higher FA in OFB than PAS sites, but this was only statistically significant in the eye (Table 2, Fig. 2). For all three traits, FA was higher in 2010–2011 samples than in 2012 samples (Table 3, Fig. 3).

**Table 1:** Descriptive statistics of FA for each of the three measured traits.

Trait	FA	ME	ICC (%)	DA
**Eye**	0.043	0.00287	93.7	t = −0.214, P = 0.831
**Pectoral Fin**	0.111	0.00692	94.1	t = 0.125, P = 0.900
**Pelvic Fin**	0.060	0.00529	91.9	t = −0.171, P = 0.865

FA and ME (measurement error, see text for details) were calculated from linear mixed model analysis. ICC (interclass correlation coefficient [FA/(FA+ME)]) is an estimate of the repeatability of FA measures. DA are tests for directional asymmetry (null of mean signed FA = 0).

**Table 2:** Mixed model ANOVA results comparing FA pre/post DWH and by location with site as a random effect.

Trait		MS	F	P
**Eye**	**Pre-Post**	5.07	20.49	<0.001 *
	**Location**	0.70	2.84	0.029 *
	Pre-Post*Location	0.08	0.34	0.667
**Pectoral**	**Pre-Post**	0.26	4.22	0.142
	**Location**	0.02	0.25	0.558
	Pre-Post*Location	0.00	0.01	0.982
**Pelvic**	**Pre-Post**	0.20	1.33	0.163
	**Location**	0.18	1.18	0.135
	Pre-Post*Location	0.21	1.40	0.308

Significance of F values assessed by maximum likelihood ratio test of nested models (see text for details).

* = significantly different (P<0.05).

**Table 3:** Mixed model ANOVA results comparing FA in 2011 and 2012 samples.

Trait		MS	F	P
**Eye**	**Year**	2.04	7.57	0.007 *
**Pectoral**	**Year**	0.45	6.76	0.010 *
**Pelvic**	**Year**	1.01	6.04	0.016 *

Significance of F values assessed by maximum likelihood ratio test of nested models (see text for details).

* = significantly different (P<0.05).

## Discussion

There was an increase in post DWH FA for one of the three traits measured and all three traits showed decreased levels of FA in 2012 compared to 2011. However, patterns in FA were not consistent with an exclusive DWH induced stress response. Only one of the three traits was different overall, and the observed higher levels of FA in OFB compared to PAS (both baseline and post DWH) was not expected. The consistent differences among the two locations may be the product of other underlying stressors that were not accounted for in the study. The abundance of *M*. *beryllina* was higher in PAS samples (mean of 117.1 +/- 16.1 SE individuals per collection) than OFB samples (31.4 +/- 7.9 SE) which may indicate PAS habitat was higher quality for this species. Both areas have a fairly substantial anthropogenic footprint which is not unusual for the northern Gulf Coast. Biloxi Bay (below OFB sites, Fig. 1) is adjacent to the cities of Gulfport and Biloxi, Mississippi, while the lower Pascagoula river areas are less populated but more exposed to pollutants from various industries in the area [46,47]. It is possible that higher baseline levels of FA in OFB reflect various stressors associated with coastal development that differ among areas. The use of museum specimens to assess FA in coastal fishes presents an opportunity to examine some impacts of coastal development over the last few decades. The U.S. Census Bureau reported the Gulf Coast as one of the most rapidly developing areas from 1960–2008. Specimens in natural history collections may be used to investigate increased baseline levels of stress in resident populations.

Fluctuating asymmetry signals were much more pronounced in the eye than either the pectoral or pelvic fins. This is consistent with findings from other FA studies on fishes. In reviewing the topic, Allenbach et al. [5] reported eye diameter as the most sensitive trait (most often showed significant FA) in fish studies while paired fin lengths were reported as substantially less sensitive. Fluctuating asymmetry has gained interest in evolutionary applications as researchers have used it as a surrogate for individual fitness [7,43,48,49]. While some progress has been made at uncovering some of the genetic underpinnings of FA, the expected relationship between FA and individual fitness is not clear, and heritability estimates of FA are minimal to no heritability at all [4,7,50]. Some research suggests that environmental canalization (developmental stability) will serve to reduce FA in traits that are under strong stabilizing selection because they are functionally very important [5,7]. Thus, environmental canalization should act to reduce phenotypic variation due to environmental inputs, especially in traits where deviations from symmetry would be expensive (such as fin asymmetry). *Menidia beryllina* is an active swimming water column fish with large pectoral fins that play an important role in locomotion. One could imagine strong selection for the size, shape, and symmetry of pectoral fins. While the eyes are also clearly important functionally, it is unlikely that small asymmetries in eye size would have the same functional cost as fin asymmetries. The likely low cost of small asymmetries in eye size may be why this has been shown to be one of the most sensitive FA traits in fishes.

There was no relationship between FA and our assay of fitness. While the measure of fitness we used (Fulton’s condition index) has been well vetted in the literature [42], our use of it in this study presents a number of potential problems. First, this index is expected to vary seasonally with changing patterns of energy allocation [28]; our fish (both pre and post DWH) were collected at various times of year, and the lack of correlation could stem from this. Other unaccounted sources of variability in condition were the age and sex of our fish. While most of the fish we used were adults (mean 49.1mm), some were immature and none of the fish were sexed. Our general approach in this study was to measure fewer traits on a larger number of fish to capture baseline FA and post DWH FA in multiple areas through time. As noted above, the expected relationship between individual fitness and measures of FA is not clear and continues to be a topic of spirited debate [7,50]. Of the three traits measured, the relationship between FA and condition was again strongest in the eye, possibly for some of the same reasons outlined above. Overall, it is likely that seasonal, ontogentic, and sex differences in energy allocation all contributed to substantial variability in our fitness metric, and the lack of a significant relationship with FA is not surprising.

Fluctuating asymmetry should be most effective at identifying stress resulting from exposure to novel toxins that would not have produced substantial stress in a species’ evolutionary history. Stressors that are either novel [7] or have only recently produced directional selection [8] are expected to generate greater levels of FA. Stressors commonly encountered may produce stabilizing selection that canalize the trait, buffering it against environmental inputs [50]. Natural oil seeps are well documented phenomena in the GOM [17,51], meaning the stress from DWH oil would be related to the magnitude of the spill and subsequent levels of exposure. The response to DWH also included the use of oil dispersants [9,11], compounds that would certainly not have produced persistent stress in the evolutionary history of *M*. *beryllina*. Similar to information regarding oilage levels at specific sites, information on where oil dispersants were applied and in what amounts are not available at spatial and temporal scales necessary to link to our collections. Ultimately, observed variability in FA could be due to some combination of oil, dispersants or other unknown stressors. *Menidia beryllina* are not known to be highly mobile fish. The closely related Atlantic Silverside (*Menidia menidia*) engages in seasonal offshore migration, and population genetic data indicate little population structuring at spatial scales pertinent to this study [52]. *Menidia beryllina* are not known to engage in any kind of large scale directional migration, and documented movement within estuaries is linked with feeding behavior [31,53].

Fluctuating asymmetry is a sensitive measure of sublethal stress and is not necessarily related to declining population levels. The post DWH samples were collected as part of broader monthly sampling of 30 sites along the MS coast. *Menidia beryllina* were consistently abundant and found in 33.1% and 30.4% of collections in 2011 and 2012, respectively. Patterns of mean abundance per collection were similar in pre and post DWH samples, and abundance was generally higher at PAS than OFB sites both pre and post DWH (Schaefer et al., in review). Thus, variability in *M*. *beryllina* abundance was consistent with what one might attribute to assemblage dynamics, and there was no obvious population decline at these sites associated with DWH. While there was no data on 2010 abundance available at these sites, other studies of fish assemblage structure and abundance for that year have reported no obvious DWH impacts [20].

The post DWH sampling that began in March 2011 yielded a surprisingly small number of fish from the 2010 year class. *Menidia beryllina* grow rapidly (0.3 mm per day) which means an individual could reach the mean size of our samples (49.1mm) in just over five months, and our minimum threshold (30 mm) in just three months. The published life history data for this species is consistent with most fish not living past one year [28], meaning most of the fish collected in our first sample (March 2011) would be young of the year. The largest fish in our post DWH sampling were collected in December and January. The fact that we randomly selected individuals (n = 20) from each collection combined with low survivorship past one year likely resulted in fewer 2010 class fish. We could have selected the largest fish from early collections in the study to maximize representation from the 2010 class, but this would have introduced other biases into the study.

## References

[1] Moller AP. Developmental instability as a general measure of stress. Adv Study Behav. 1998;27: 181–213. 9562900

[2] Clarke GM. Relationships between developmental stability and fitness: Application for conservation biology. Conserv Biol. 1995;9: 18–24.

[3] Klingenberg CP. A developmental perspective on developmental instability: theory, models, and mechanisms In: Polak M, editor. Developmental Instability: Causes and Consequences. Oxford University Press; 2003 pp. 14–34.

[4] Nijhout HF, Davidowitz G. Developmental perspectives on phenotypic variation, canalization, and fluctuating asymmetry In: Polak M, editor. Developmental Instability Causes and Consequences. Oxford University Press; 2003 pp. 3–13.

[5] Allenbach D. Fluctuating asymmetry and exogenous stress in fishes: a review. Rev Fish Biol Fish. 2011;21: 355–376.

[6] Leary RF, Allendorf FW. Fluctuating asymmetry as an indicator of stress: Implications for conservation biology. Trends Ecol Evol. 1989;4: 214–217. 10.1016/0169-5347(89)90077-3 21227354

[7] Graham JH, Raz S, Hel-Or H, Nevo E. Fluctuating asymmetry: methods, theory, and applications. Symmetry. 2010;2: 466–540.

[8] De Coster G, Van Dongen S, Malaki P, Muchane M, Alcántara-Exposito A, Matheve H, et al Fluctuating asymmetry and environmental stress: understanding the role of trait history. PLoS ONE. 2013;8: e57966 10.1371/journal.pone.0057966 23472123 PMC3589457

[9] Reddy CM, Arey JS, Seewald JS, Sylva SP, Lemkau KL, Nelson RK, et al Composition and fate of gas and oil released to the water column during the Deepwater Horizon oil spill. Proc Natl Acad Sci. 2012;109: 20229–20234. 10.1073/pnas.1101242108 21768331 PMC3528605

[10] Crone TJ, Tolstoy M. Magnitude of the 2010 Gulf of Mexico oil leak. Science. 2010;330: 634 10.1126/science.1195840 20929734

[11] Peterson CH, Anderson SS, Cherr GN, Ambrose RF, Anghera S, Bay S, et al A tale of two spills: novel science and policy implications of an emerging new oil spill model. BioScience. 2012;62: 461–469. 10.1525/bio.2012.62.5.7

[12] Williams R, Gero S, Bejder L, Calambokidis J, Kraus SD, Lusseau D, et al Underestimating the damage: interpreting cetacean carcass recoveries in the context of the Deepwater Horizon/BP incident. Conserv Lett. 2011;4: 228–233. 10.1111/j.1755-263X.2011.00168.x

[13] McCrea-Strub A, Kleisner K, Sumaila UR, Swartz W, Watson R, Zeller D, et al Potential impact of the Deepwater Horizon oil spill on commercial fisheries in the Gulf of Mexico. Fisheries. 2011;36: 332–336. 10.1080/03632415.2011.589334

[14] Edwards BR, Reddy CM, Camilli R, Carmichael CA, Longnecker K, Mooy BASV. Rapid microbial respiration of oil from the Deepwater Horizon spill in offshore surface waters of the Gulf of Mexico. Environ Res Lett. 2011;6: 035301 10.1088/1748-9326/6/3/035301

[15] Silliman BR, Koppel JVD, McCoy MW, Diller J, Kasozi GN, Earl K, et al Degradation and resilience in Louisiana salt marshes after the BP–Deepwater Horizon oil spill. Proc Natl Acad Sci. 2012;109: 11234–11239. 10.1073/pnas.1204922109 22733752 PMC3396483

[16] Dubansky B, Whitehead A, Miller JT, Rice CD, Galvez F. Multitissue molecular, genomic, and developmental effects of the Deepwater Horizon oil spill on resident gulf killifish (*Fundulus grandis*). Environ Sci Technol. 2013;47: 5074–5082. 10.1021/es400458p 23659337 PMC3746035

[17] Murawski SA, Hogarth WT, Peebles EB, Barbeiri L. Prevalence of external skin lesions and polycyclic aromatic hydrocarbon concentrations in Gulf of Mexico fishes, post-Deepwater Horizon. Trans Am Fish Soc. 2014;143: 1084–1097. 10.1080/00028487.2014.911205

[18] Whitehead A, Dubansky B, Bodinier C, Garcia TI, Miles S, Pilley C, et al Science Applications in the Deepwater Horizon Oil Spill Special Feature: Genomic and physiological footprint of the Deepwater Horizon oil spill on resident marsh fishes. Proc Natl Acad Sci. 2011;100: 20298–20302. doi:http://www.pnas.org/cgi/doi/10.1073/pnas.1109545108 10.1073/pnas.1109545108 PMC3528528 21949382

[19] Brette F, Machado B, Cros C, Incardona JP, Scholz NL, Block BA. Crude oil impairs cardiac excitation-contraction coupling in fish. Science. 2014;343: 772–776. 10.1126/science.1242747 24531969

[20] Fodrie FJ, Heck KL. Response of coastal fishes to the Gulf of Mexico oil disaster. PLoS ONE. 2011;6: e21609 10.1371/journal.pone.0021609 21754992 PMC3130780

[21] Moody RM, Cebrian J, Heck KL. Interannual recruitment dynamics for resident and transient marsh species: evidence for a lack of impact by the macondo oil spill. PLoS ONE. 2013;8: e58376 10.1371/journal.pone.0058376 23516467 PMC3596379

[22] Teal JM, Howarth RW. Oil spill studies: A review of ecological effects. Environ Manage. 1984;8: 27–43. 10.1007/BF01867871

[23] Lee DS, Gilbert CR, Hocutt CH, Jenkins RE, McAllister DE, Stauffer JR. Atlas of North American Freshwater Fishes. Raleigh, NC: North Carolina Biological Survey; 1980.

[24] Gundersen DT, Kristanto SW, Curtis LR, Al-Yakoob SN, Metwally MM, Al-Ajmi D. Subacute toxicity of the water-soluble fractions of Kuwait crude oil and partially combusted crude oil on *Menidia beryllina* and *Palaemonetes pugio* . Arch Environ Contam Toxicol. 1996;31: 1–8. 8687984 10.1007/BF00203901

[25] Middaugh DP, Chapman PJ, Shelton ME. Responses of embryonic and larval inland silversides, *Menidia beryllina*, to a water-soluble fraction formed during biodegradation of artificially weathered Alaska North Slope crude oil. Arch Environ Contam Toxicol. 1996;31: 410–419. 8854836 10.1007/BF00212681

[26] Solangi MA, Overstreet RM. Histopathological changes in two estuarine fishes, *Menidia beryllina* (Cope) and *Trinectes maculatus* (Bloch and Schneider), exposed to crude oil and its water-soluble fractions. J Fish Dis. 1982;5: 13–35. 10.1111/j.1365-2761.1982.tb00453.x

[27] Bengtson DA. Resource partitioning by *Menidia menidia* and *Menidia beryllina* (Osteichthyes: Atherinidae). Mar Ecol Prog Ser. 1984;18: 21–30.

[28] Middaugh DP, Hemmer MJ. Reproductive ecology of the inland silverside, *Menidia beryllina*, (Pisces: Atherinidae) from Blackwater Bay, Florida. Copeia. 1992;1992: 53–61. 10.2307/1446535

[29] Huber M, Bengtson DA. Effects of photoperiod and temperature on the regulation of the onset of maturation in the estuarine fish *Menidia beryllina* (Cope) (Atherinidae). J Exp Mar Biol Ecol. 1999;240: 285–302. 10.1016/S0022-0981(99)00064-7

[30] Ross ST. Inland Fishes of Mississippi Oxford, MS: University Press of Mississippi; 2000.

[31] Gleason TR, Bengtson DA. Size-selective mortality of inland silversides: Evidence from otolith microstructure. Trans Am Fish Soc. 1996;125: 860–873.

[32] Peterson MS, Ross ST. Dynamics of littoral fishes and decapods along a coastal river-estuarine gradient. Estuar Coast Shelf Sci. 1991;33: 467–483.

[33] Peterson MS, Weber MR, Partyka ML, Ross ST. Integrating *in situ* quantitative geographic information tools and size-specific, laboratory-based growth zones in a dynamic river-mouth estuary. Aquat Conserv Mar Freshw Ecosyst. 2007;17: 602–618.

[34] Peterson MS, Slack WT, Woodley CM. The occurrence of non-indigenous nile tilapia, *Oreochromis niloticus* (Linnaeus) in coastal Mississippi, USA: Ties to aquaculture and thermal effluent. Wetlands. 2005;25: 112–121.

[35] Mariano AJ, Kourafalou VH, Srinivasan A, Kang H, Halliwell GR, Ryan EH, et al On the modeling of the 2010 Gulf of Mexico oil spill. Dyn Atmospheres Oceans. 2011;52: 322–340. 10.1016/j.dynatmoce.2011.06.001

[36] Almeida D, Almodovar A, Nicola GG, Elvira B. Fluctuating asymmetry, abnormalities and parasitism as indicators of environmental stress in cultured stocks of goldfish and carp. Aquaculture. 2008;279: 120–125.

[37] Young WP, Frenyea K, Wheeler PA, Thorgaard GH. No increase in developmental deformities or fluctuating asymmetry in rainbow trout (*Oncorhynchus mykiss*) produced with cryopreserved sperm. Aquaculture. 2009;289: 13–18.

[38] Eriksen M, Espmark Ã, Poppe T, Braastad B, Salte R, Bakken M. Fluctuating asymmetry in farmed Atlantic salmon (*Salmo salar*) juveniles: also a maternal matter? Environ Biol Fishes. 2008;81: 87–99.

[39] Merila J, Björklund M. Fluctuating asymmetry and measurement error. Syst Biol. 1995;44: 97–101.

[40] Katsanevakis S. Modelling fish growth: Model selection, multi-model inference and model selection uncertainty. Fish Res. 2006;81: 229–235.

[41] Jakob EM, Marshall SD, Uetz GW. Estimating fitness: a comparison of body condition indices. Oikos. 1996;77: 61–67. 10.2307/3545585

[42] Weber LP, Higgins PS, Carlson RI, Janz DM. Development and validation of methods for measuring multiple biochemical indices of condition in juvenile fishes. J Fish Biol. 2003;63: 637–658.

[43] Kristoffersen JB, Magoulas A. Fluctuating asymmetry and fitness correlations in two *Engraulis encrasicolus* populations. J Fish Biol. 2009;75: 2723–2736. 10.1111/j.1095-8649.2009.02473.x 20738519

[44] Godet JP, Demuynck S, Waterlot C, Lemière S, Souty-Grosset C, Douay F, et al Fluctuating asymmetry analysis on *Porcellio scaber*(Crustacea, Isopoda) populations living under metals-contaminated woody habitats. Ecol Indic. 2012;23: 130–139.

[45] R Development Core. R: A language and environment for statistical computing Vienna, Austria: R Foundation for Statistical Computing; 2012.

[46] Lytle TF, Lytle JS. Pollutant Transport in Mississippi Sound. Biloxi, Mississippi; 1985 p. 124. Report No.: MSAGS-82-038.

[47] Partyka ML, Peterson MS. Habitat quality and salt-marsh species assemblages along an anthropogenic estuarine landscape. J Coast Res. 2008;246: 1570–1581. 10.2112/07-0937.1

[48] Morris MR, Rios-Cardenas O, Lyons SM, Scarlett Tudor M, Bono LM. Fluctuating asymmetry indicates the optimization of growth rate over developmental stability. Funct Ecol. 2012;26: 723–731. 10.1111/j.1365-2435.2012.01983.x

[49] Dongen SV. Fluctuating asymmetry and developmental instability in evolutionary biology: past, present and future. J Evol Biol. 2006;19: 1727–1743. 10.1111/j.1420-9101.2006.01175.x 17040371

[50] Debat V, David P. Mapping phenotypes: canalization, plasticity and developmental stability. Trends Ecol Evol. 2001;16: 555–561. 10.1016/S0169-5347(01)02266-2

[51] Macdonald IR, Guinasso NL, Ackleson SG, Amos JF, Duckworth R, Sassen R, et al Natural oil slicks in the Gulf of Mexico visible from space. J Geophys Res Oceans. 1993;98: 16351–16364. 10.1029/93JC01289

[52] Roark SA, Kelble MA, Nacci D, Champlin D, Coiro L, Guttman SI. Population genetic structure and tolerance to dioxin-like compounds of a migratory marine fish (*Menidia menidia*) at polychlorinated biphenyl–contaminated and reference sites. Environ Toxicol Chem. 2005;24: 726–732. 15779775 10.1897/03-688.1

[53] Wurtsbaugh W, Li H. Diel migrations of (*Menidia beryllina*) in relation to the distribution of its prey in a large eutrophic lake. Limnol Ocean. 1985;30: 565476.

